# Does ceiling‐fan use modify circulating markers of heat‐related stress in bed‐resting older adults?

**DOI:** 10.1113/EP094380

**Published:** 2026-11-14

**Authors:** Glen P. Kenny, Kelli E. McCormick, Fergus K. O'Connor, James J. McCormick

**Affiliations:** ^1^ Human and Environmental Physiology Research Unit, School of Human Kinetics University of Ottawa Ottawa Ontario Canada; ^2^ Institute of the Environment University of Ottawa Ottawa Canada; ^3^ Interdisciplinary School of Health Sciences, Faculty of Health Sciences University of Ottawa Ottawa Ontario Canada; ^4^ Clinical Epidemiological Program Ottawa Hospital Research Institute Ottawa Ontario Canada; ^5^ School of Allied Health, Sport and Social Work Griffith University Southport Queensland Australia; ^6^ Allied Health Research Collaborative The Prince Charles Hospital Chermside Queensland Australia

**Keywords:** ageing, gastrointestinal barrier, heat stress, indoor overheating, inflammation, renal injury

## Abstract

Ceiling fans are a low‐energy strategy for reducing heat strain, but their ability to protect against subclinical cellular and organ stress is unknown. We examined circulating stress biomarkers in 20 older adults (12 females; median age 71 years), who completed randomized 8 h exposures to 31°C and 45% relative humidity while remaining reclined beneath a ceiling fan delivering 0 m s^−1^ (control) or 1.5 m s^−1^ (fan). Venous blood was collected before and after each exposure and analysed for serum intestinal fatty acid‐binding protein (IFABP), soluble cluster of differentiation 14 (sCD14), kidney injury molecule‐1, neutrophil gelatinase‐associated lipocalin, tumour necrosis factor‐α, interleukin‐6 and C‐reactive protein. Core temperature and heart rate were measured continuously. Biomarker responses were assessed using baseline‐adjusted linear mixed‐effects models with Holm correction for multiple testing. Fan use lowered peak core temperature and heart rate by 0.2°C (*P *< 0.001) and 5 beats min^−1^ (*P* = 0.030), respectively. The adjusted difference in IFABP change between control and fan was 344 pg mL^−1^ [95% confidence interval: 102–586] (unadjusted *P* = 0.005; Holm‐adjusted *P* = 0.035). Across conditions, the estimated changes in IFABP and sCD14 were 240 pg mL^−1^ [95% confidence interval: 11–468] (Holm‐adjusted *P* = 0.240) and 0.57 ng mL^−1^ [95% confidence interval: 0.10–1.03] (Holm‐adjusted *P* = 0.119), respectively. No other biomarker comparisons remained significant after Holm adjustment. Ceiling‐fan use at 31°C attenuated the IFABP response in bed‐resting older adults despite modest effects on core temperature and heart rate. These exploratory findings suggest that fan‐mediated protection might extend beyond changes captured by conventional physiological measures, but confirmation in prospectively powered studies incorporating direct measures of gastrointestinal permeability is required.

## Abstract (highlights)

- **What is the central question?**
  Does ceiling‐fan use protect against circulating markers of intestinal, renal and inflammatory stress in bed‐resting older adults during daylong exposure to indoor overheating?
- **What is the main finding and its importance?**
  Ceiling‐fan use attenuated the intestinal fatty acid‐binding protein response despite producing only modest reductions in core temperature and heart rate. At 31°C, where airflow should favour heat dissipation, ceiling fans might provide a selective biomarker benefit not fully captured by conventional physiological measures, although effects on other biomarkers were not detected.

## 1 INTRODUCTION

Climate change is increasing exposure to extreme heat. In 2024, adults older than 65 years experienced a record global average of 20.8 heatwave days per person (Romanello et al., 2025). During these events, people without effective air‐conditioning may experience prolonged indoor overheating. Older adults are disproportionately vulnerable because ageing and common chronic conditions can impair heat dissipation and cardiovascular regulation, while limited mobility may restrict behavioural responses, such as relocating to a cooler space or repositioning near a cooling device (Kenny et al., 2024). This is especially relevant for people who remain in bed for much of the day, for whom a large proportion of the body surface is insulated by the bed and unavailable for heat exchange. The consequences of heat exposure can also extend into the night, because higher nighttime temperatures are associated with shorter sleep duration and poorer sleep quality, with older adults among the populations most vulnerable to heat‐related sleep disturbance (Chevance et al., 2024).

Electric fans are a low‐cost, low‐energy intervention that increase air movement across the skin. When air temperature is below skin temperature, fan‐generated airflow enhances both convective and evaporative heat loss. As air temperature rises above skin temperature, however, airflow increases convective heat gain, and its net effect depends on whether enhanced sweat evaporation can offset that additional heat gain. Fans therefore should not be considered universally protective at high temperatures. In older adults, fan cooling becomes less effective above ∼33°C and can worsen heat strain above 40°C, particularly in very hot, dry conditions (Meade et al., 2024; O'Connor et al., 2025). Consistent with this guidance, fan use reduced cardiac strain in older adults at 38°C and 60% relative humidity but increased cardiac strain at 45°C and 15% relative humidity (Chaseling et al., 2024). At 31°C and 45% relative humidity, skin temperature generally remains above air temperature, and fan‐generated airflow should favour both dry and evaporative heat loss. Nevertheless, in our previous study of bed‐resting older adults in these conditions, ceiling‐fan use lowered peak core temperature and heart rate by 0.2°C and 5 beats min^−1^, respectively (O'Connor et al., 2026). These effects approached but did not reach predefined thresholds of clinical relevance. Even with fan use, peak core temperature and heart rate increased from baseline by ∼0.9°C and 22 beats min^−1^, respectively. Thus, ceiling‐fan use provided measurable but incomplete physiological protection.

Core temperature and heart rate, however, might not fully characterize the biological consequences of prolonged heat exposure. Heat strain can initiate a graded, multisystem response involving splanchnic hypoperfusion and intestinal epithelial injury, exposure to microbial products and innate immune activation, renal tubular stress and systemic inflammation (Cramer et al., 2022; Lim, 2018; Schlader et al., 2022). The selected biomarker panel represented these related but distinct pathways: intestinal fatty acid‐binding protein (IFABP) as a marker of enterocyte injury; soluble cluster of differentiation 14 (sCD14) as an index of innate immune responses to microbial products; kidney injury molecule‐1 (KIM‐1) and neutrophil gelatinase‐associated lipocalin (NGAL) as markers of renal tubular stress or injury; and tumour necrosis factor‐α (TNF‐α), interleukin‐6 (IL‐6) and C‐reactive protein (CRP) as indicators of systemic inflammatory signalling and the acute‐phase response. Collectively, the panel included organ‐associated markers of intestinal and renal stress or injury alongside broader markers of immune and inflammatory activation. Because these processes differ in sensitivity and time course, a modest heat exposure may alter an early epithelial marker without producing a coordinated response across the entire panel.

Recent evidence supports this possibility. Older adults exhibit greater increases in circulating IFABP than younger adults during extreme passive heat exposure (Foster et al., 2023), and controlled hyperthermia can increase gastrointestinal permeability and indices of kidney dysfunction or injury (McKenna et al., 2025). Extreme heat exposure can also impair kidney function even when overt clinical injury is not apparent (McKenna et al., 2024). In our daylong indoor‐overheating studies, IFABP showed an emerging elevation at 31°C and a clearer increase at 36°C, whereas TNF‐α, IL‐6 and CRP did not increase in parallel (Lee, Meade et al., 2025). These findings suggest that organ‐associated markers of stress or injury might respond at indoor temperatures, whereas changes in conventional physiological outcomes, such as core temperature, and downstream systemic markers, such as inflammation, remain comparatively modest.

Previous work at 36°C showed that pedestal‐fan airflow did not attenuate intestinal, renal or inflammatory biomarker responses in seated older adults (Lee, McCarthy et al., 2025). Whether this applies to ceiling‐fan use at 31°C, an environment in which airflow should be biophysically favourable, and to older adults remaining supine is unknown. Previous reports from this randomized crossover trial and the same cohort of 20 older adults described the physiological and perceptual outcomes. The primary physiological analysis found small but clear reductions in core temperature and heart rate with fan use (O'Connor et al., 2026). A separate analysis of perceptual outcomes found lower heat‐related symptom burden and thermal discomfort, although total mood disturbance was unchanged (McGarr et al., 2026). Whether these physiological and perceptual benefits were accompanied by altered biological stress remained unresolved. We therefore examined whether ceiling‐fan use modified circulating markers of intestinal epithelial injury, immune activation, renal tubular stress or injury and systemic inflammation during 8 h of simulated indoor overheating in bed‐resting older adults. We hypothesized that fan use would attenuate heat‐related changes in one or more of these circulating biomarkers.

## 2 MATERIALS AND METHODS

### 2.1 Ethical approval and study design

This exploratory biomarker analysis represents the third report from the same randomized crossover trial examining ceiling‐fan use during simulated indoor overheating. It used stored serum samples collected from the same participant cohort; the physiological and perceptual outcomes from the trial have been reported previously (McGarr et al., 2026; O'Connor et al., 2026). The study was approved by the University of Ottawa Health Sciences and Science Research Ethics Board (H‐11‐21‐7575) and conformed to the *Declaration of Helsinki*. The trial was prospectively registered on ClinicalTrials.gov (NCT06142890). Written informed consent was obtained from all volunteers prior to their participation. All work was performed at the University of Ottawa.

### 2.2 Screening and participants

Twenty community‐dwelling adults aged 65–85 years completed the study (12 females; median [interquartile range] age, 71 [68–73] years). Details of participant characteristics can be found in Table 1. A between‐condition difference of ≥0.2°C in peak core temperature was predefined as clinically relevant, and 17 participants were required to detect this effect with >90% power. No separate a priori sample‐size calculation was performed for the exploratory biomarker outcomes (O'Connor et al., 2026).

**TABLE 1:** Physical characteristics of enrolled participants.

Variable	All participants (*n* = 20)
Age, years	71 (68–73)
Females, *n* (%)	12 (60)
Males, *n* (%)	8 (40)
Height, cm	167 (157–173)
Mass, kg	73.9 (61.3–81.1)
Body mass index, kg m^−2 ^ ^a^	25.1 (23.6–28.0)
Body surface area, m^2^ ^b^	1.9 (1.6–1.9)
Smoking status^c^	
Never, *n* (%)	14 (70)
Past, *n* (%)	6 (30)
Habitual physical activity, min week^−1 ^ ^d^	150 (120–393)
Types of physical activity, *n* (%)^d^	
Walking	14 (70)
Jogging, biking, swimming, rowing	3 (15)
Organized sports	3 (15)
Peak aerobic power, mL kg^−1^ min^−1 ^ ^e^	23 (21–25)
ACSM peak aerobic power percentile, %^e^	10 (4–20)
Using prescribed medications, *n* (%)^f^	15 (75)
Heat vulnerability‐linked medications, *n* (%)^f^	
Antihypertensives	9 (45)
Antidepressants	1 (5)
Metformin	1 (5)
Haemoglobin A_1c_, %	5.6 (5.3–5.9)

*Note*: Values are the median (interquartile range) or number of participants (%).

^a^ Body mass index was calculated as weight (in kilograms) divided by the square of height (in metres).

^b^ Body surface area was estimated using the Du Bois equation: 0.20247 × height (in metres)^0.725^ × mass (in kg)^0.425^.

^c^ Smoking status was determined via participant self‐report. Prospective participants were excluded if they were currently smoking. All past smokers quit ≥15 years prior to participation.

^d^ Participant self‐reported physical activity level was determined via the Canadian Society for Exercise Physiology (2017) Get Active Questionnaire. The types of physical activity performed were determined via the Kohl Physical Activity Questionnaire.

^e^ Peak aerobic power of prospective participants was assessed during an incremental semi‐recumbent cycling test to volitional fatigue. Percentile derived from the American College of Sports Medicine (ACSM).

^f^ Participant prescription medication use was determined via self‐report. Some participants reported taking medications that have been suggested to increase heat vulnerability (e.g., antidepressants) or are associated with health conditions known to reduce heat tolerance (e.g., type 2 diabetes, heart disease). Reported medications included: antihypertensives (e.g., angiotensin‐converting enzyme inhibitors, angiotensin II receptor blockers, diuretics, calcium channel blockers; *n* = 9), antidepressants (e.g., serotonin and noradrenaline reuptake inhibitors, selective serotonin reuptake inhibitors, *n* = 1), metformin (*n* = 1), statins (*n* = 8), topical creams/ointments (*n* = 2), corticosteroids (*n* = 4), bronchodilators (*n* = 1); and medications treating hypoactive thyroid (*n* = 4), occasional pain (e.g., for headaches and joint pain; *n* = 3) and osteoporosis (*n* = 4).

Volunteers completed the Canadian Society for Exercise Physiology (2017) Get Active Questionnaire, with activity types detailed using the Kohl Physical Activity Questionnaire (Kohl et al., 1988). The screening visit included medical and medication histories, resting blood pressure, height, body mass and an incremental semi‐recumbent cycling test to volitional fatigue. Peak oxygen uptake and cardiac responses were assessed during the test, with continuous 12‐lead ECG monitoring by a certified exercise physiologist. Individuals were excluded for current smoking, physical restrictions or unstable disease that made participation inadvisable, relevant cardiac abnormalities or symptoms during screening, heat acclimatization, or peak oxygen uptake above the 50th percentile of age‐ and sex‐specific normative values. Full eligibility criteria, medication use and screening procedures are reported in the primary physiological report (O'Connor et al., 2026).

### 2.3 Experimental protocol

Participants completed two experimental trials in randomized order. They were asked to avoid strenuous activity and alcohol for 24 h and excessive caffeine for 12 h, to consume 500 mL of water the evening before and the morning of each trial, and to replicate their pretrial activity, sleep and dietary patterns. Hydration was confirmed on arrival from urine specific gravity; values ≥1.025 were corrected with 500 mL of water and reassessed after ∼30 min. Trials began between 06:00 and 09:00 h. After instrumentation, participants rested for ∼60 min in a temperate room (∼22°C) while baseline measurements were collected.

Participants then completed 8 h at 31°C and 45% relative humidity while wearing shorts and a light shirt and resting in a semi‐reclined position on a medical bed. No blankets, duvets or other coverings were placed over participants during the exposure. A commercially available ceiling fan positioned directly above the bed delivered an airspeed of either 0 m s^−1^ (CON) or ∼1.5 m s^−1^, measured at participant level (FAN). Water (∼22°C) was available ad libitum, and participants could consume a self‐provided lunch during the first 4 h. Except for a prespecified cardiac autonomic test battery conducted during hour 6, participants remained reclined throughout the exposure. The battery included heart rate variability assessment during paced breathing, quantified using the standard deviation of normal‐to‐normal intervals (SDNN) and root mean square of successive differences (RMSSD), and two supine‐to‐standing tests assessing the 30:15 heart‐rate ratio, systolic blood‐pressure response and symptoms of orthostatic intolerance. Orthostatic‐intolerance symptoms recorded during the battery have been reported previously (O'Connor et al., 2026). Venous blood was collected before the exposure in a thermoneutral environment and at the end of the exposure in the heated environment.

### 2.4 Physiological and perceptual measurements

Core temperature was measured continuously using a thermocouple probe inserted ∼12 cm beyond the anal sphincter (Mon‐a‐therm General Purpose Temperature Probe, Mallinckrodt Medical Inc., St Louis, MO, USA). Heart rate was derived from beat‐to‐beat RR intervals recorded using a three‐lead ECG (Bio Amp, ADInstruments, Colorado Springs, CO, USA). Thermal discomfort was assessed at baseline and hourly using a visual analog scale converted to values from −50 (extremely comfortable) through 0 (neutral) to +50 (extremely uncomfortable). Heat‐related symptoms and mood state were assessed at baseline and end‐exposure using the validated 68‐item Environmental Symptoms Questionnaire (ESQ‐IV) and 40‐item Profile of Mood States questionnaire (POMS‐40), respectively. Full processing and questionnaire scoring procedures are reported elsewhere (McGarr et al., 2026; O'Connor et al., 2026).

### 2.5 Venous blood collection and biochemical analysis

Participants rested for ∼30 min before each sample collection. Venous blood was obtained before and at the end of each 8 h exposure into serum‐separator and K_2_EDTA tubes (BD Vacutainer, Becton Dickinson, Franklin Lakes, NJ, USA). Serum‐separator tubes were left to clot for ∼15 min and were then centrifuged at 1380*g* for 10 min. Serum was aliquoted and stored at −80°C until analysis. Haemoglobin and haematocrit were measured in duplicate from EDTA‐treated blood using an automated haematology analyser (DxH 500, Beckman Coulter, Brea, CA, USA). Percentage change in plasma volume was estimated from haemoglobin and haematocrit (Dill & Costill, 1974).

Serum samples were analysed in duplicate using commercially available sandwich ELISAs (R&D Systems, Minneapolis, MN, USA) for IFABP (DY3078), sCD14 (DY383), IL‐6 (DY206), TNF‐α (DY210), CRP (DY1707), NGAL (DY1757) and KIM‐1 (DY1750). Samples were diluted 1:5 in PBS containing 1% bovine serum albumin, except CRP, which was diluted 1:250. Assays were performed according to the manufacturer's instructions. Absorbance was measured at 450 nm with correction at 570 nm using a SynergyMX plate reader (BioTek, Winooski, VT, USA). Inter‐ and intra‐assay coefficients of variation were <8%.

### 2.6 Statistical analysis

All protein concentrations were corrected for changes in plasma volume before analysis. The change from baseline to the end of heat exposure was analysed separately for each biomarker using linear mixed‐effects models. Condition was modelled as a fixed effect with two levels (CON and FAN), with the corresponding baseline concentration included as a continuous covariate. A random intercept was included for each participant. Estimated marginal mean changes from baseline across conditions and contrasts between CON and FAN were derived from each model. Between‐condition effects are reported as the adjusted mean difference (CON minus FAN) with 95% confidence intervals. To account for multiple testing, the Holm procedure was applied separately to the seven overall changes from baseline and the seven between‐condition contrasts because these represent distinct families of statistical tests. Both unadjusted and Holm‐adjusted *P*‐values are reported, with statistical significance determined using the Holm‐adjusted *P*‐values threshold of *P* ≤ 0.05. All analyses were performed using R v.4.2.0 (R Foundation for Statistical Computing, Vienna, Austria) within the RStudio integrated development environment (Posit Software, PBC, Boston, MA, USA). Linear mixed‐effects models were fitted using the lme4 and lmerTest packages, and estimated marginal means and between‐condition contrasts were obtained using the emmeans package (Bates et al., 2015).

## 3 RESULTS

### 3.1 Physiological responses

The thermoregulatory and cardiovascular outcomes are reported fully elsewhere (O'Connor et al., 2026) and are summarized here for context. Core temperature increased by a mean (SD) of 1.1 (0.5)°C during CON and 0.9 (0.3)°C during FAN, reaching peak values of 37.8 (0.2)°C and 37.6 (0.2)°C, respectively. Peak core temperature was 0.2°C lower with FAN [95% confidence interval: 0.1–0.3] (*P* < 0.001). Peak and end‐exposure heart rates were also lower with FAN than CON by 5 and 2 beats min^−1^, respectively (both *P* = 0.030). Plasma volume increased by 3.3% (7.5%) during CON and 3.8% (6.8%) during FAN. The magnitude of this increase did not differ between conditions (FAN minus CON adjusted difference, 0.4 percentage points [95% confidence interval: −3.1 to 3.9]; *P* = 0.81).

### 3.2 Perceptual responses

Perceptual outcomes are reported fully elsewhere (McGarr et al., 2026; O'Connor et al., 2026). End‐exposure thermal discomfort was 9.0 mm lower with FAN than CON [95% confidence interval: 4.2–13.8] (*P* < 0.001). After adjustment for baseline score and core‐temperature area under the curve, ESQ‐IV total symptom scores were 1.3‐fold higher during CON than FAN [95% confidence interval: 1.1–1.7] (*P* = 0.015). POMS‐40 total mood disturbance did not differ between conditions (*P* = 0.460).

### 3.3 Intestinal epithelial injury and immune activation

Across conditions, the estimated change in IFABP from baseline was 240 pg mL^−1^ [95% confidence interval: 11–468] (unadjusted *P* = 0.040; Holm‐adjusted *P* = 0.240). The IFABP response differed between conditions, with an adjusted difference in change between CON and FAN of 344 pg mL^−1^ [95% confidence interval: 102–586] (unadjusted *P* = 0.005; Holm‐adjusted *P* = 0.035; Figure 1a,b). Across conditions, the estimated change in sCD14 was 0.57 ng mL^−1^ [95% confidence interval: 0.10–1.03] (unadjusted *P* = 0.017; Holm‐adjusted *P* = 0.119), with no clear between‐condition difference (CON minus FAN: 0.09 ng mL^−1^ [95% confidence interval: −0.44 to 0.62]; unadjusted *P* = 0.738; Holm‐adjusted *P* > 0.999; Figure 1c,d).

**FIGURE 1:**
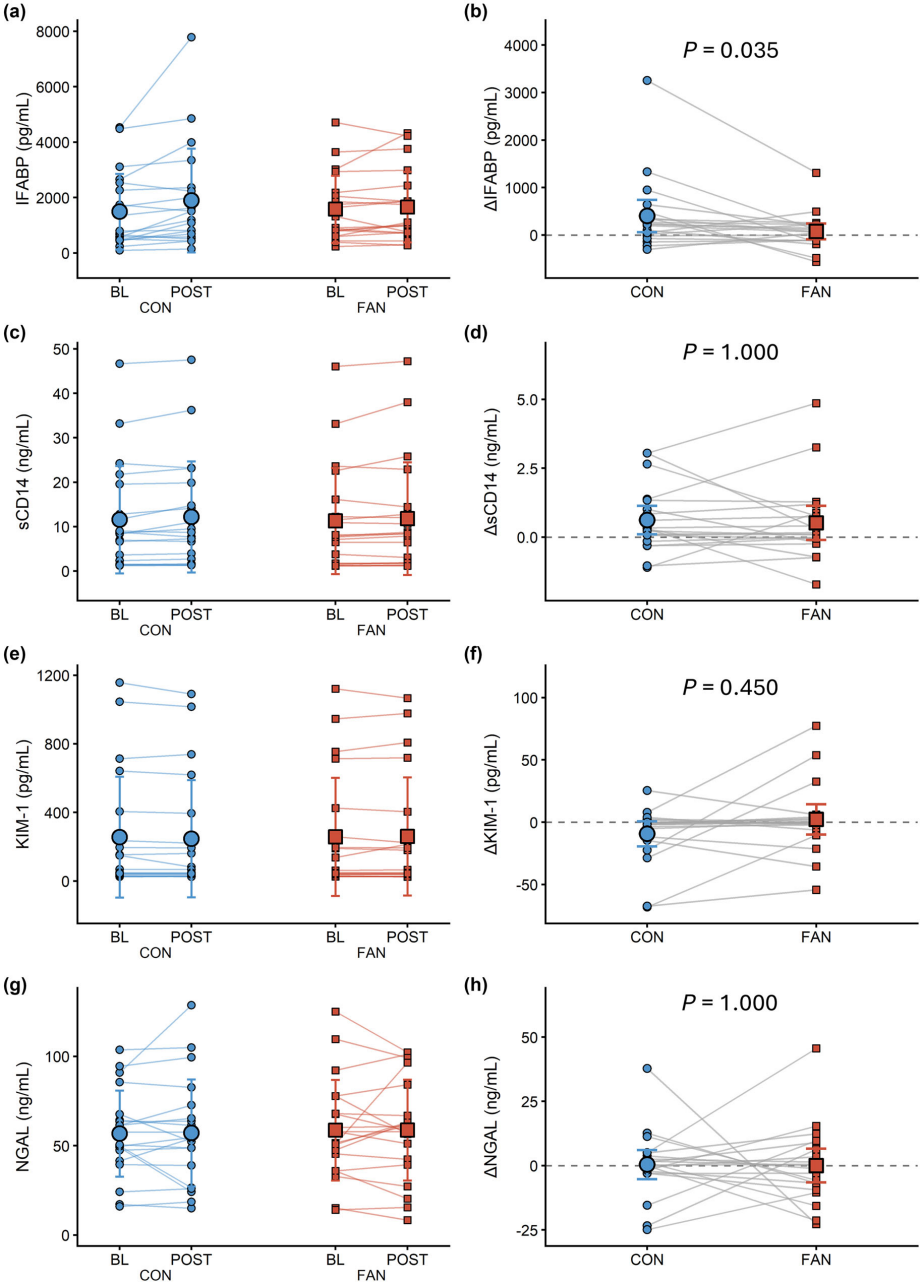
Circulating markers of intestinal epithelial injury, immune activation and renal injury before (BL) and after (POST) 8 h of simulated indoor overheating at 31°C and 45% relative humidity without (control; CON) or with a ceiling fan delivering ∼1.5 m s^−1^ (FAN). Absolute concentrations and individual changes are shown for intestinal fatty acid‐binding protein (IFABP; a,b), soluble cluster of differentiation 14 (sCD14; c,d), kidney injury molecule‐1 (KIM‐1; e,f) and neutrophil gelatinase‐associated lipocalin (NGAL; g,h). Circles represent CON and squares FAN. Small symbols and connecting lines represent individual participant responses. In panels a, c, e and g, large symbols and error bars represent the group mean and SD. In panels b, d, f and h, large symbols and error bars represent the mean change from baseline and 95% confidence interval. *P*‐values in panels b, d, f and h represent baseline‐adjusted between‐condition contrasts derived from linear mixed‐effects models, with the Holm procedure applied across FAN versus CON biomarker comparisons.

### 3.4 Renal injury and systemic inflammation

KIM‐1 did not change clearly from baseline across conditions (−3.47 pg mL^−1^ [95% confidence interval: −12.40 to 5.47]; unadjusted *P* = 0.447; Holm‐adjusted *P* > 0.999), and the between‐condition estimate was imprecise (CON minus FAN: −11.67 pg mL^−1^ [95% confidence interval: −24.53 to 1.19]; unadjusted *P* = 0.075; Holm‐adjusted *P* = 0.450; Figure 1e,f). NGAL also remained unchanged across conditions (0.23 ng mL^−1^ [95% confidence interval: −4.16 to 4.63]; unadjusted *P* = 0.917; Holm‐adjusted *P* > 0.999), with no clear effect of FAN (CON minus FAN: 0.32 ng mL^−1^ [95% confidence interval: −8.47 to 9.12]; unadjusted *P* = 0.942; Holm‐adjusted *P* > 0.999; Figure 1g,h).

No clear changes from baseline were detected across conditions for TNF‐α (−0.31 pg mL^−1^ [95% confidence interval: −0.95 to 0.33]; unadjusted *P* = 0.349; Holm‐adjusted *P* > 0.999), IL‐6 (−0.23 pg mL^−1^ [95% confidence interval: −0.70 to 0.24]; unadjusted *P* = 0.341; Holm‐adjusted *P* > 0.999) or CRP (−0.011 µg mL^−1^ [95% confidence interval: −0.033 to 0.012]; unadjusted *P* = 0.356; Holm‐adjusted *P* > 0.999). Corresponding between‐condition differences for TNF‐α, IL‐6 and CRP were −0.31 pg mL^−1^ [95% confidence interval: −1.60 to 0.97] (unadjusted *P* = 0.632; Holm‐adjusted *P* > 0.999), 0.32 pg mL^−1^ [95% confidence interval: −0.62 to 1.26] (unadjusted *P* = 0.504; Holm‐adjusted *P* > 0.999) and 0.011 µg mL^−1^ [95% confidence interval: −0.031 to 0.053; unadjusted *P* = 0.616; Holm‐adjusted *P* > 0.999), respectively (Figure 2).

**FIGURE 2:**
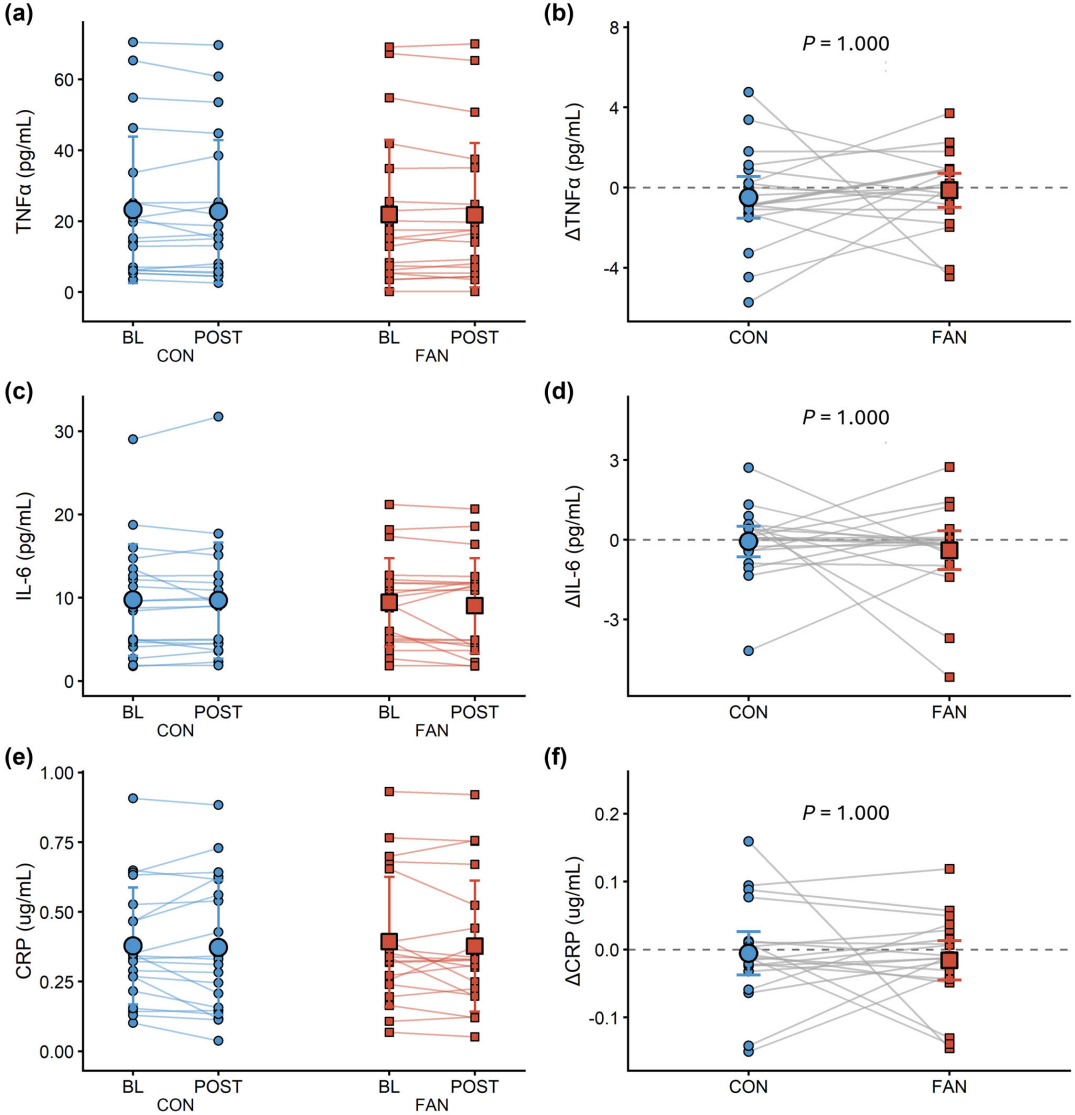
Circulating inflammatory markers before (BL) and after (POST) 8 h of simulated indoor overheating at 31°C and 45% relative humidity without (control; CON) or with a ceiling fan delivering ∼1.5 m s^−1^ (FAN). Absolute concentrations and individual changes are shown for tumour necrosis factor‐α (TNF‐α; a,b), interleukin‐6 (IL‐6; c,d) and C‐reactive protein (CRP; e,f). Circles represent CON and squares FAN. Small symbols and connecting lines represent individual participant responses. In panels a, c and e, large symbols and error bars represent the group mean and SD. In panels b, d and f, large symbols and error bars represent the mean change from baseline and 95% confidence interval. *P*‐values in panels b, d and f represent baseline‐adjusted between‐condition contrasts derived from linear mixed‐effects models, with the Holm procedure applied across FAN versus CON biomarker comparisons.

## 4 DISCUSSION

This study identified a selective rather than uniform biomarker response to daylong indoor overheating. The estimated overall changes in IFABP and sCD14 were positive but did not remain statistically significant after Holm adjustment. However, the IFABP response differed between conditions and was attenuated by ceiling‐fan use. KIM‐1, NGAL, TNF‐α, IL‐6 and CRP showed no clear overall or fan‐related changes. Thus, evidence of fan‐related biochemical protection was limited to the between‐condition IFABP effect and accompanied the modest reductions in core temperature and heart rate observed in the primary physiological analysis (O'Connor et al., 2026). However, because most other biomarkers did not change during either exposure, their null between‐condition contrasts should not be interpreted as evidence that fan use failed to protect these pathways. Rather, this heat exposure might not have elicited changes in these markers that could be modified detectably by the fan.

IFABP is abundant in mature enterocytes and is released into the systemic circulation following epithelial injury. Extreme passive heat and controlled hyperthermia increase IFABP or gastrointestinal permeability, with some evidence that older adults are more susceptible than younger adults (Foster et al., 2023; McKenna et al., 2025). Heat‐related redistribution of blood flow away from the splanchnic circulation, combined with direct thermal effects, can produce intestinal ischaemia and epithelial disruption. Loss of barrier integrity can facilitate microbial product translocation and immune activation, forming part of the pathophysiological cascade of severe heat illness (Lim, 2018; Schlader et al., 2022). The present exposures produced substantially less hyperthermia than these extreme models, yet IFABP still increased across conditions. This complements evidence of an emerging IFABP elevation after 8 h at 31°C and a larger response at 36°C in older adults (Lee, Meade et al., 2025), suggesting that intestinal epithelial stress might begin during prolonged exposure to indoor temperatures commonly encountered in homes without effective cooling.

The attenuation of IFABP with FAN contrasts with earlier findings that pedestal‐fan airflow at 2 or 4 m s^−1^ did not modify IFABP during seated exposure to 36°C (Lee, McCarthy et al., 2025). The environmental and postural differences might have contributed to the contrasting findings. At 36°C, air temperature exceeded typical skin temperature, increasing convective heat gain even as airflow promoted evaporation. At 31°C, the air‐to‐skin gradient should permit additional convective heat loss, making a protective effect more likely. Ceiling‐fan airflow also acts from above, while much of the posterior body surface is insulated by the bed. The finding that a benefit of fan use was detectable despite the reduced body surface area available for fan‐enhanced heat exchange is particularly relevant to individuals with limited mobility who cannot readily reposition themselves or relocate to a cooler environment.

The mechanism linking fan use to lower IFABP cannot be determined from circulating biomarkers alone. Even a 0.2°C reduction in peak core temperature and a 5 beat min^−1^ reduction in peak heart rate could lessen cumulative splanchnic circulatory strain or direct epithelial heat exposure. Such small whole‐body differences might matter if the epithelial response has a lower activation threshold or a steep temperature‐response relationship. However, splanchnic blood flow, gastrointestinal permeability, endotoxin and microbial translocation were not measured, hence this interpretation remains mechanistic speculation rather than evidence of preserved barrier function.

Several features of the exposure might help to explain why the remaining markers did not differ. First, 31°C at rest produced less hyperthermia than studies using 36°C–44°C, exercise, or a controlled increase of ∼2°C in core temperature (Cramer et al., 2022; Foster et al., 2023; McKenna et al., 2025). The magnitude of heat stress might have been sufficient to produce a detectable increase in IFABP and increase sCD14, but insufficient to elicit detectable changes in TNF‐α or IL‐6. CRP is a hepatic acute‐phase protein with a slower response than these cytokines, making an immediate end‐exposure sample particularly unlikely to capture a delayed increase. Second, water was available ad libitum, which might have limited dehydration and associated renal perfusion stress. This might help to explain the absence of KIM‐1 and NGAL responses. Correction for changes in plasma volume also reduced the influence of exposure‐related haemoconcentration on circulating biomarker concentrations. Circulating KIM‐1 and NGAL provide an incomplete view of renal stress, and NGAL is not entirely kidney specific. Urinary biomarkers or direct measures of kidney function might be more sensitive in this setting (McKenna et al., 2024). Future studies incorporating repeated blood and urine sampling could provide a more complete assessment of the timing and magnitude of renal responses to prolonged indoor heat exposure. Finally, sampling only before and immediately after exposure could miss transient cytokine peaks or renal and acute‐phase responses that emerge during recovery. Accordingly, the null estimates should be viewed as specific to the exposure intensity, biological matrix and sampling time, rather than evidence that these pathways are irrelevant to indoor heat exposure.

The accompanying physiological and perceptual findings help to place the biomarker pattern in context. Ceiling‐fan use produced small reductions in core temperature and heart rate (O'Connor et al., 2026) and reduced heat‐related symptom burden and thermal discomfort (McGarr et al., 2026), although it did not improve total mood disturbance. The perceptual outcomes are best viewed as complementary indicators of participants’ subjective experience of the heat exposure rather than mechanistic correlates of the circulating biomarkers. Thermal discomfort and symptom burden reflect this subjective experience, whereas IFABP reflects a distinct organ‐specific response. Their parallel attenuation with fan use therefore represents separate responses to the same cooling intervention, not evidence of a direct relationship between perceptual change and IFABP. Conversely, continued elevations in core temperature and heart rate and the absence of clear fan‐related differences in total mood disturbance or the other biomarkers argue against interpreting the fan as complete protection.

### 4.1 Perspective

In bed‐resting older adults exposed to 31°C, ceiling‐fan use provided modest physiological and perceptual relief and attenuated the IFABP response. It reduced thermal and cardiovascular strain, lessened heat‐related symptoms and thermal discomfort, and attenuated a circulating marker of enterocyte injury, yet it did not measurably alter total mood disturbance, the sCD14 response or the assessed renal and systemic inflammatory markers. This selective response indicates that core temperature and heart rate alone might not capture the full range of biological responses to a cooling intervention, but it should not be interpreted as broad protection from heat‐related stress. Although not assessed in the present study, modelling suggests that, when environmental conditions are suitable, using fans to delay the initiation of air‐conditioning might reduce cooling‐related energy demand and greenhouse‐gas emissions (Malik et al., 2022). Air‐conditioning or access to a cooler environment remains necessary when fan use provides insufficient cooling or might worsen heat strain. Future trials should combine repeated blood and urine sampling with direct measures of intestinal permeability, renal function and hydration to determine whether attenuation of circulating IFABP reflects preserved intestinal barrier integrity and is accompanied by reduced subsequent immune or organ stress.

### 4.2 Limitations

Several limitations warrant consideration. The biomarker outcomes were exploratory and were not included in the original sample‐size calculation, which was based on the predefined between‐condition difference in peak core temperature. Holm adjustment was applied separately to the overall changes from baseline and the between‐condition contrasts. Nevertheless, the study was not specifically powered to detect differences in biomarker outcomes, and non‐significant comparisons should not be interpreted as evidence of no effect or equivalence. Although the IFABP between‐condition effect remained statistically significant after Holm adjustment, it should be confirmed in a prospectively designed study with a sample size determined for biomarker outcomes. IFABP is a circulating marker of enterocyte injury and does not measure gastrointestinal permeability directly. Biomarkers were sampled only before and immediately after exposure, hence transient or delayed responses might have been missed. Finally, the controlled laboratory setting and relatively healthy sample limit generalizability to frailer or clinically bedbound adults, who might have greater heat vulnerability.

## 5 CONCLUSION

In conclusion, ceiling‐fan use during daylong exposure to 31°C attenuated the circulating IFABP response, a marker of intestinal epithelial injury, in bed‐resting older adults, despite producing only modest reductions in core temperature and heart rate. These exploratory findings suggest that the benefits of ceiling‐fan use might extend beyond changes captured by conventional physiological measures in conditions biophysically favourable to airflow, but do not demonstrate preservation of intestinal barrier integrity or broad protection against heat‐related biological stress. Confirmation using direct measures of gastrointestinal permeability and studies in frailer, mobility‐limited adults is warranted.

## AUTHOR CONTRIBUTIONS

All experimental procedures and biochemical analyses were performed at the Human and Environmental Physiology Research Unit, University of Ottawa. Glen P. Kenny and Fergus K. O'Connor conceived and designed the study. Glen P. Kenny performed the initial literature searches. Kelli E. McCormick, Fergus K. O'Connor and James J. McCormick collected the data. James J. McCormick performed the statistical analyses and prepared the figures. Glen P. Kenny drafted the manuscript. All authors interpreted the findings, revised the manuscript critically for important intellectual content, approved the final version and agree to be accountable for all aspects of the work in ensuring that questions related to the accuracy or integrity of any part of the work are appropriately investigated and resolved. All persons designated as authors qualify for authorship, and all those who qualify are listed.

## CONFLICT OF INTEREST

Glen P. Kenny reported receiving funding from the Canadian Institutes of Health Research and/or Health Canada. No other disclosures were reported.

## GENERATIVE AI STATEMENT

No generative AI tools were used to draft the content of this manuscript. Google AI technologies were used solely for literature search retrieval and information verification.

## Data Availability

Deidentified participant data and analytical code are available from the corresponding author (gkenny@uottawa.ca) upon reasonable request, without any time restrictions. Permission for reuse of data must be obtained in writing from the corresponding author.

## References

[eph70559-bib-0001] Bates, D. , Maechler, M. , Bolker, B. , & Walker, S. (2015). Fitting linear mixed‐effects models using lme4. Journal of Statistical Software, 67(1), 1–48.

[eph70559-bib-0019] Canadian Society for Exercise Physiology . (2017). Get active questionnaire.

[eph70559-bib-0002] Chaseling, G. K. , Vargas, N. T. , Hospers, L. , Barry, H. , Harwood, A. , Graham, C. , Bartlett, A. A. , Debray, A. , Lynch, G. , Capon, A. , Crandall, C. G. , Fiatarone Singh, M. , Mavros, Y. , Bi, P. , Nigam, A. , Chabot‐Blanchet, M. , Gagnon, D. , & Jay, O. (2024). Simple strategies to reduce cardiac strain in older adults in extreme heat. New England Journal of Medicine, 391(18), 1754–1756. 10.1056/NEJMc2407812 PMC12121688 39504526

[eph70559-bib-0003] Chevance, G. , Minor, K. , Vielma, C. , Campi, E. , O'Callaghan‐Gordo, C. , Basagana, X. , Ballester, J. , & Bernard, P. (2024). A systematic review of ambient heat and sleep in a warming climate. Sleep Medicine Reviews, 75, 101915. 10.1016/j.smrv.2024.101915 38598988

[eph70559-bib-0004] Cramer, M. N. , Gagnon, D. , Laitano, O. , & Crandall, C. G. (2022). Human temperature regulation under heat stress in health, disease, and injury. Physiological Reviews, 102(4), 1907–1989. 10.1152/physrev.00047.2021 PMC9394784 35679471

[eph70559-bib-0005] Dill, D. B. , & Costill, D. L. (1974). Calculation of percentage changes in volumes of blood, plasma, and red cells in dehydration. Journal of Applied Physiology, 37(2), 247–248. 10.1152/jappl.1974.37.2.247 4850854

[eph70559-bib-0006] Foster, J. , McKenna, Z. J. , Atkins, W. C. , Jarrard, C. P. , & Crandall, C. G. (2023). Aging increases enterocyte damage during a 3‐hour exposure to very hot and dry heat: A preliminary study. Biology, 12(8), 1088. 10.3390/biology12081088 PMC10451985 37626974

[eph70559-bib-0007] Kenny, G. P. , Tetzlaff, E. J. , Journeay, W. S. , Henderson, S. B. , & O'Connor, F. K. (2024). Indoor overheating: A review of vulnerabilities, causes, and strategies to prevent adverse human health outcomes during extreme heat events. Temperature, 11(3), 203–246. 10.1080/23328940.2024.2361223 PMC11346563 39193048

[eph70559-bib-0008] Kohl, H. W. , Blair, S. N. , Paffenbarger, R. S., Jr. , Macera, C. A. , & Kronenfeld, J. J. (1988). A mail survey of physical activity habits as related to measured physical fitness. American Journal of Epidemiology, 127(6), 1228–1239. 10.1093/oxfordjournals.aje.a114915 3369421

[eph70559-bib-0009] Lee, B. J. , Meade, R. D. , Davey, S. L. , Thake, C. D. , McCormick, J. J. , King, K. E. , & Kenny, G. P. (2025). Effects of daylong exposure to indoor overheating on enterocyte damage and inflammatory responses in older adults: A randomized crossover trial. Applied Physiology, Nutrition and Metabolism, 50, 1–7. 10.1139/apnm-2024-0368 39869857

[eph70559-bib-0010] Lee, B. J. , McCarthy, T. , O'Connor, F. , Davey, S. L. , Thake, C. D. , McCormick, J. J. , King, K. E. , Boulay, P. , Meade, R. D. , & Kenny, G. P. (2025). Effect of pedestal fan use on serum stress biomarkers in older adults exposed to simulated daylong indoor overheating. Physiological Reports, 13(12), e70390. 10.14814/phy2.70390 PMC12181687 40542541

[eph70559-bib-0011] Lim, C. L. (2018). Heat sepsis precedes heat toxicity in the pathophysiology of heat stroke‐A new paradigm on an ancient disease. Antioxidants, 7(11), 149. 10.3390/antiox7110149 PMC6262330 30366410

[eph70559-bib-0012] Malik, A. , Bongers, C. , McBain, B. , Rey‐Lescure, O. , Dear, R. , Capon, A. , Lenzen, M. , & Jay, O. (2022). The potential for indoor fans to change air conditioning use while maintaining human thermal comfort during hot weather: An analysis of energy demand and associated greenhouse gas emissions. Lancet Planetary Health, 6(4), e301–e309. 10.1016/S2542-5196(22)00042-0 35397218

[eph70559-bib-0013] McGarr, G. W. , O'Connor, F. K. , & Kenny, G. P. (2026). Effects of ceiling fans on self‐reported symptoms and mood‐state in bed‐resting older adults exposed to indoor overheating. Journal of the American Geriatrics Society, 74(6), 1703–1708. 10.1111/jgs.70388 PMC13266453 41833559

[eph70559-bib-0014] McKenna, Z. J. , Atkins, W. C. , Foster, J. , Belval, L. N. , Watso, J. C. , Jarrard, C. P. , & Crandall, C. G. (2024). Kidney function biomarkers during extreme heat exposure in young and older adults. Journal of the American Medical Association, 332(4), 333–335. 10.1001/jama.2024.9845 PMC11197017 38913388

[eph70559-bib-0015] McKenna, Z. J. , Atkins, W. C. , Wallace, T. , Jarrard, C. P. , Crandall, C. G. , & Foster, J. (2025). Gastrointestinal permeability and kidney injury risk during hyperthermia in young and older adults. Experimental Physiology, 110(1), 79–92. 10.1113/EP092204 PMC11689130 39417775

[eph70559-bib-0016] Meade, R. D. , Notley, S. R. , Kirby, N. V. , & Kenny, G. P. (2024). A critical review of the effectiveness of electric fans as a personal cooling intervention in hot weather and heatwaves. Lancet Planetary Health, 8(4), e256–e269. 10.1016/S2542-5196(24)00030-5 38580427

[eph70559-bib-0017] O'Connor, F. K. , Kenny, G. P. , & Meade, R. D. (2025). Staying cool during heat exposure. CMAJ: Canadian Medical Association Journal, 197(40), E1343. 10.1503/cmaj.250951 PMC12638113 41285467

[eph70559-bib-0018] O'Connor, F. K. , Meade, R. D. , Janetos, K. T. , Li‐Maloney, C. , Sigal, R. J. , Boulay, P. , & Kenny, G. P. (2026). Effect of ceiling fans on core temperature in bed‐resting older adults exposed to indoor overheating. Journal of the American Geriatrics Society, 74(1), 200–204. 10.1111/jgs.70109 PMC12774295 41014079

[eph70559-bib-0020] Romanello, M. , Walawender, M. , Hsu, S. C. , Moskeland, A. , Palmeiro‐Silva, Y. , Scamman, D. , Smallcombe, J. W. , Abdullah, S. , Ades, M. , Al‐Maruf, A. , Ameli, N. , Angelova, D. , Ayeb‐Karlsson, S. , Ballester, J. , Basagana, X. , Bechara, H. , Beggs, P. J. , Cai, W. , Campbell‐Lendrum, D. , & Costello, A. (2025). The 2025 report of the Lancet countdown on health and climate change: Climate change action offers a lifeline. Lancet, 406(10521), 2804–2857. 10.1016/S0140-6736(25)01919-1 41175887

[eph70559-bib-0021] Schlader, Z. J. , Davis, M. S. , & Bouchama, A. (2022). Biomarkers of heatstroke‐induced organ injury and repair. Experimental Physiology, 107(10), 1159–1171. 10.1113/EP090142 PMC9529995 35654394

